# A Review on MR Image Intensity Inhomogeneity Correction

**DOI:** 10.1155/IJBI/2006/49515

**Published:** 2006-08-07

**Authors:** Zujun Hou

**Affiliations:** Biomedical Imaging Lab., Singapore Bioimaging Consortium, 30 Biopolis Street, Matrix #07-01, 138671, Singapore

## Abstract

Intensity inhomogeneity (IIH) is often encountered in MR imaging, and a number of techniques have been devised to correct this artifact. This paper attempts to review some of the recent developments in the mathematical modeling of IIH field. Low-frequency models are widely used, but they tend to corrupt the low-frequency components of the tissue. Hypersurface models and statistical models can be adaptive to the image and generally more stable, but they are also generally more complex and consume more computer memory and CPU time. They are often formulated together with image segmentation within one framework and the overall performance is highly dependent on the segmentation process. Beside these three popular models, this paper also summarizes other techniques based on different principles. In addition, the issue of quantitative evaluation and comparative study are discussed.


Hindawi Publishing Corporation
International Journal of Biomedical Imaging
Volume 2006, Article ID 49515, Pages 1–11
DOI 10.1155/IJBI/2006/49515

### A Review on MR Image Intensity Inhomogeneity Correction

**Zujun Hou**

*Biomedical Imaging Lab., Singapore Bioimaging Consortium, 30 Biopolis Street, Matrix #07-01, Singapore 138671*

Received 11 October 2005; Revised 18 January 2006; Accepted 17 February 2006

Intensity inhomogeneity (IIH) is often encountered in MR imaging, and a number of techniques have been devised to correct this artifact. This paper attempts to review some of the recent developments in the mathematical modeling of IIH field. Low-frequency models are widely used, but they tend to corrupt the low-frequency components of the tissue. Hypersurface models and statistical models can be adaptive to the image and generally more stable, but they are also generally more complex and consume more computer memory and CPU time. They are often formulated together with image segmentation within one framework and the overall performance is highly dependent on the segmentation process. Beside these three popular models, this paper also summarizes other techniques based on different principles. In addition, the issue of quantitative evaluation and comparative study are discussed.

Copyright © 2006 Zujun Hou. This is an open access article distributed under the Creative Commons Attribution License, which permits unrestricted use, distribution, and reproduction in any medium, provided the original work is properly cited.

### 1. INTRODUCTION

With the frequent application of the magnetic resonance (MR) imaging method to clinical diagnosis, automatic analysis of the acquired images using techniques from computer vision and pattern recognition has received considerable attention. In developing such computer-aided diagnosis tools, a commonly encountered problem is to correct the intensity inhomogeneity (IIH) in MR images.

The IIH (also termed as the intensity nonuniformity, the bias field, or the gain field in the literature) usually refers to the slow, nonanatomic intensity variations of the same tissue over the image domain. It can be due to imaging instrumentation (such as radio-frequency nonuniformity, static field inhomogeneity, etc.) or the patient movement [1–5]. This artifact is particularly severe in MR images captured by surface coils. Two real MR images with severe IIH artifact are shown in Figure 1(a), where one can see that the intensity varies significantly for the pixels of the same tissue and the intensity values overlap markedly between the pixels of the different tissues. For comparison, the IIH corrected images by a surface fitting technique [6] are given in Figure 1(b), from which the improvement in image quality is clearly visible. The estimated IIH maps are given in Figure 1(c).

Let $\mathbf{x}$ denote the measured intensity and $\mathbf{x}'$ the true intensity. Then the most popular model in describing the IIH effect is

$$\mathbf{x} = \boldsymbol{\alpha}\mathbf{x}' + \boldsymbol{\xi}, \tag{1}$$

where $\boldsymbol{\alpha}$ denotes the IIH effect and $\boldsymbol{\xi}$ the noise. Notation of bold letters refers to 2D or 3D MR data. Figure 2 displays the widely used BrainWeb [7] simulated MR images, where on Figure 2(a) is the original image, on Figure 2(b) the image with IIH artifact, on Figure 2(c) the image with noise, and on Figure 2(d) the image with both IIH artifact and noise. From Figure 2, one can see the visual difference resulting from the IIH artifact and the noise.

To simplify the computation, one often ignores the noise and takes the logarithmic transform of intensity

$$y_i = \log x_i = \log x_i' + \log \alpha_i = y_i' + \beta_i, \tag{2}$$

where $x_i$ is the intensity at voxel $i$ ($i = 1, \ldots, n$). Here, to avoid numerical problems, care should be taken for those pixels/voxels with low intensities, which are usually excluded from computation.

In general, the presence of IIH can significantly reduce the accuracy of image segmentation and registration, hence decreasing the reliability of subsequent quantitative measurement. A number of techniques have been proposed to deal with this issue. In general, if a map of the IIH in the image domain (Figure 1(c) for instance) is known or can be estimated, then it is simple to correct the IIH by division in (1) or subtraction in the log-domain (2). One can obtain the IIH map from measurement in vivo [8–15], typically of a uniform phantom, [16–20], which often requires extra measurement (and increases the scanning time) or needs additional hardware which may not be readily available in some clinical departments. Also there are theoretical modeling approaches

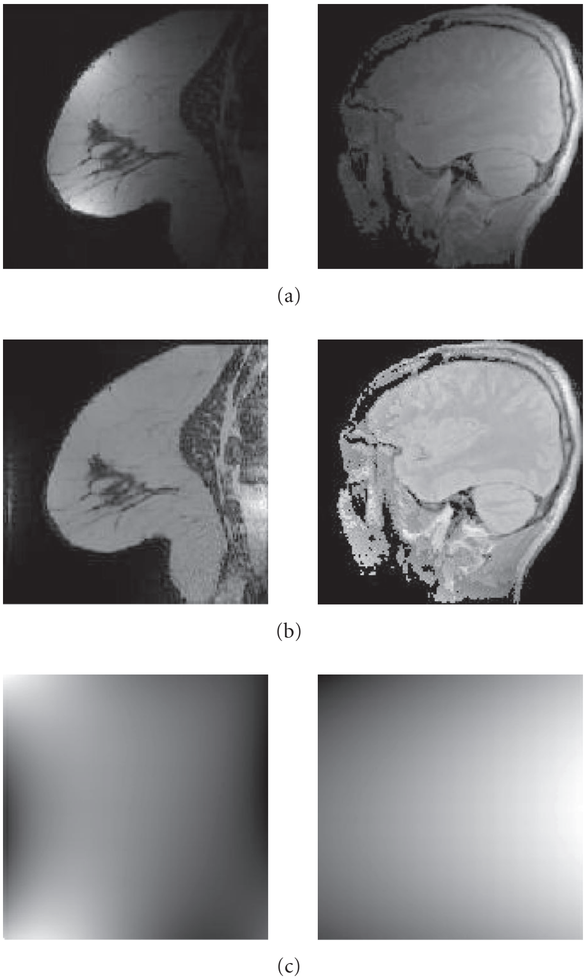

Figure 1: Sample MR images with severe intensity inhomogeneity: original images (a), corrected images (b), and estimated inhomogeneity maps (c).

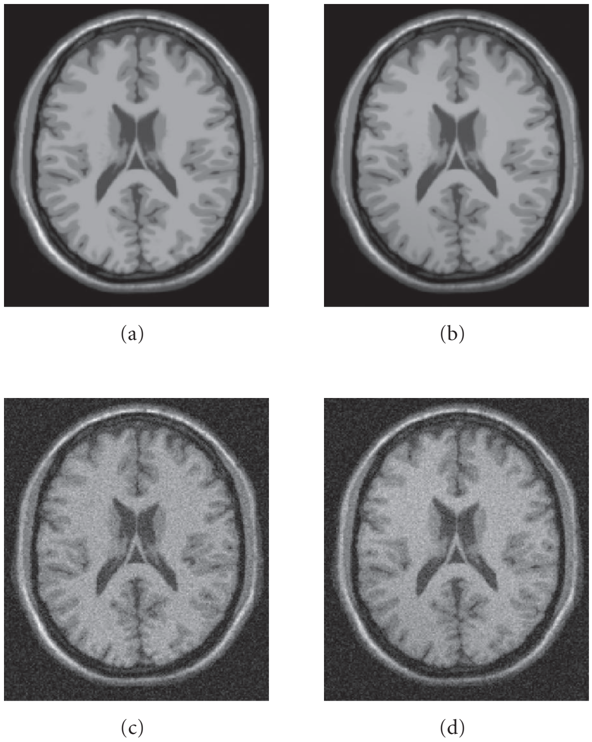

Figure 2: BrainWeb simulated images: original image (a), image with 40% inhomogeneity (b), image with 9% noise (c), and image with both artifacts (d).

[21–28] to approximate the IIH map. However, due to the complexity that causes the IIH, it is difficult to model the IIH under a variety of imaging conditions. In particular, the object-induced IIH is hard to be accounted for by phantom study or theoretical modeling.

More often, the IIH map is derived retrospectively from the image data alone. A number of research efforts have been put in this direction and many techniques have been proposed. Popular mathematical models for IIH description can be classified as follows:

(1) low-frequency model, which assumes the IIH to constitute low-frequency components in frequency domain and the IIH map can be recovered by lowpass filtering;
(2) hypersurface model, which fits the IIH map by a smooth functional, whose parameters are usually obtained using regression;
(3) statistical model, which assumes the IIH to be a random variable or a random process and the IIH map can be derived through statistical estimation;
(4) others, which are based on different principles, and sometimes without explicit assumptions on the IIH field.

With this in mind, the IIH correction methods are categorized into lowpass filtering, statistical modeling, surface fitting and others, which are detailed, respectively, in the following sections.

For an early literature review, interested readers are referred to [29], where an evaluation of the IIH correction effect for brain tumor segmentation is also reported. This paper attempts to summarize the recent progress and focus will be on mathematical modeling for IIH removal. Nevertheless, it is by no means an exhaustive summary. For simplicity, the description will be on single-channel data only.

### 2. LOWPASS FILTERING

Since the IIH is slowly varying in the image domain, its spectrum in frequency domain will be concentrated in the low-frequency end. Therefore the IIH could be separated from the true image by a lowpass filter, $\mathcal{L}$. After lowpass filtering in log-domain, one would approximately have

$$\mathcal{L}\{\mathbf{y}\} \approx \beta. \tag{3}$$

This procedure to correct the IIH is similar to the homomorphic filtering in digital image processing for the correction of illumination inhomogeneity [30]. In fact, (1) easily reminds one of the illumination-reflectance model in optical imaging [30], where the artifact from the illumination inhomogeneity is often termed “shading” in the literature. Thus, techniques for shading correction, such as the homomorphic filtering, can be adopted for IIH removal and the converse also holds. An investigation of applying IIH correction methods to deal with the shading problem in microscopic images has been carried out in [31].

Due to their simplicity and efficiency in implementation, lowpass filtering methods have been widely used [30–43]. For a summary, interested readers are referred to [42]. Also in [42], the impact of filter width on IIH correction was investigated and it was found that these methods should be used with care to avoid intensity distortion and artificial artifacts in the corrected images. Basically, for MR images, due to the overlapping spectrum between the patient data and the IIH, the effectiveness of most conventional lowpass filtering in removing the IIH is generally quite limited.

Luo et al. [44] presented a technique to recover low-frequency components which correspond to anatomic structure and are lost during the lowpass filtering. The method expresses the signal with a linear combination of singularity functions. The higher-frequency components are assumed to be less affected by the IIH and are used to reconstruct the true image, after which the ratio between the observed and estimated image is used for IIH map approximation.

Recently, lowpass filtering methods have been extended using the wavelet transform [45, 46] and were shown to be effective in removing IIH in images acquired by surface coils and phase array coils. Compared with usual lowpass filtering methods, the multiresolution analysis allows one to select an optimal scale from which the approximate band in the wavelet transform domain is used for estimating the IIH map.

In [47], an improvement of a lowpass filtering method [43] was presented. The method varies the filter kernel size to minimize the segmentation error. The idea is generally similar to [45, 46] in addressing IIH correction from the scale space, but differs in the criterion to determine the optimal scale.

### 3. SURFACE FITTING

Since the inhomogeneity field is slowly varying, it is natural to approximate the IIH by a parametric smooth functional [6, 48–58]. Very often, the parameter estimation is linked to image segmentation. In this way, the two different problems, IIH correction and image segmentation, are formulated in one framework and solved simultaneously. Alternatively, the parameter searching can be guided through the variation of some global image feature in an iterative process. A typical example is to minimize the entropy of gray-level histogram.

### *3.1. Segmentation*

A large category of surface fitting approaches search the parameters by fitting with respect to a set of tissue points encoding information about the IIH. Let $I = \{1, \dots, n\}$ index the voxel coordinates of brain tissue. Then, in order to determine the parameters of this functional, one needs to find/segment a set of voxels $S_I \subseteq I$ which convey information about the IIH map. Among these methods, the essential difference lies in the identification of $S_I$, and hence decoding the IIH information from $S_I$.

Dawant et al. [48] proposed to manually select $S_I$ such that they belong to the same type of tissue. As a result, the intensity variation among these voxels can largely be attributed to IIH. However, expert supervision to select points is time consuming and error prone, especially for volume data. Wang et al. [59] has presented an automated method for generating the reference points.

Meyer et al. [49] employed the LCJ method [60] to preliminarily segment the image and then fit a smooth functional over the segmented image. The LCJ segmentation method assumes the image to be piecewise smooth and requires that the different objects are well separated at the boundaries, which is quite stringent in practice, particularly when image quality is poor due to perturbation such as noise, partial volume artifact, or IIH. Beside that, not every clinical department can afford the computer cost to run the parallel LCJ algorithm.

Liew and Yan [58] approximated the IIH as a stack of *B*-spline surfaces with continuity constraints across slices. The estimation of IIH interwines with a fuzzy *c*-means clustering process. In [61, 62], segmentation that utilizes local scale as homogeneous criteria has been presented and applied to IIH correction.

When a statistical classifier, such as Gaussian classifier or random field modeling, is exploited [50, 53, 63], the process is similar to the parameter estimation in Section 4, where the parameters are associated with a probability distribution and can be estimated with common statistical estimation methods such as maximum-likelihood estimation.

### *3.2. Entropy minimization*

As a frequently used criterion to characterize the intensity distribution of an image, entropy has been employed to design algorithms for image restoration, thresholding, or classification [64, 65]. Also, it has been utilized to quantify the image property with IIH present and guide the parameter searching for IIH removal [54–57].

It is assumed that the intensity distribution of the original image is multimodal, and the presence of IIH causes the intensity overlapping between objects. Figure 3 shows the histograms of brain tissue in a BrainWeb-simulated image (Figure 3(a)) and the head image in Figure 1 (Figure 3(b)). On Figure 3(a), the solid line is the histogram without inhomogeneity, where the modes corresponding to different tissues are very distinctive. With the presence of IIH (dashed-dotted line), the valleys between different modes are markedly flattened. For the head image with severe IIH, the histogram (Figure 3(b)) is so flat that the modes corresponding to the gray matter and the white matter are difficult to distinguish. The flattening of the histogram leads to the increase in the entropy of the image, therefore, the IIH

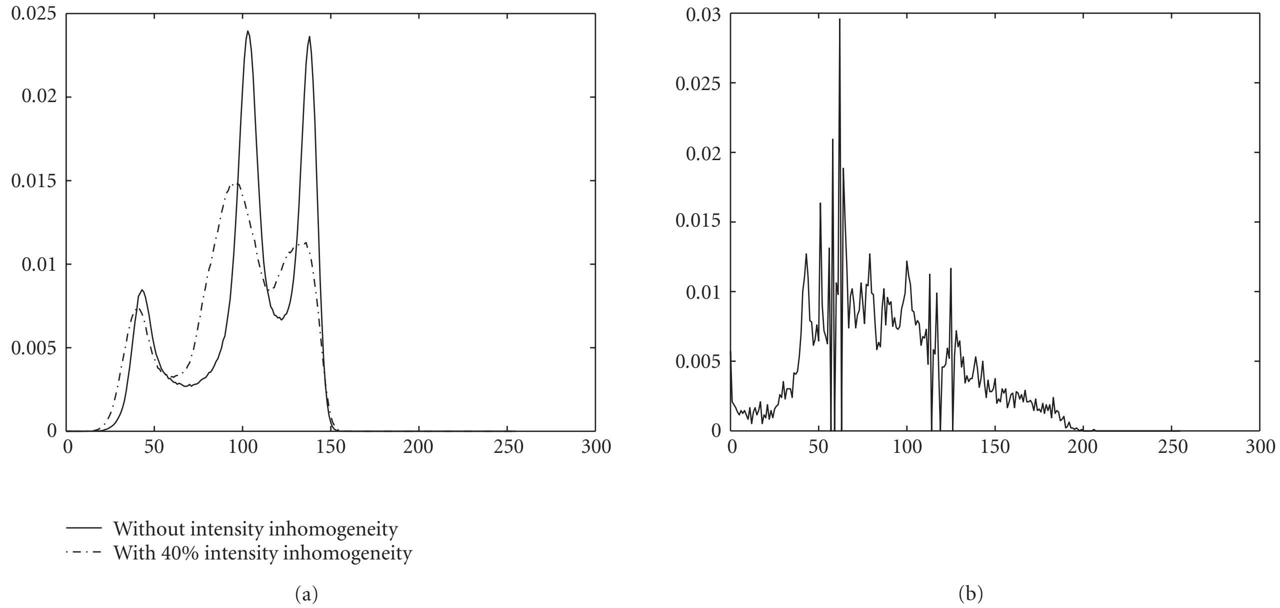

FIGURE 3: Histogram of brain tissue with the presence of intensity inhomogeneity. (a) Corresponds to a BrainWeb-simulated image: without intensity inhomogeneity (solid line) and with 40% intensity inhomogeneity (dashed-dotted line), and on (b) the head image (Figure 1).

correction can be achieved through searching the parameter space of the IIH model such that the entropy of the image is reduced. It should be pointed out that direct minimization on the entropy would lead to the null field [55, 66]. To avoid this pitfall, constraints over the solution space are necessary. Mangin [55] constrained the solution to minimize the distance between the mean values of the restored and the original image. In [57], the restored image was constrained to have the same mean value as the original one.

Evidently, other quantities relevant to image features, variance for example, can also be applicable in a similar fashion. Again, constraints upon the solution space are necessary.

### 4. STATISTICAL MODELING

The statistical methods [67–71] may assume that the IIH follows a distribution, the Gaussian distribution for example, or model the IIH as a random process, such as the Markov random field.

### 4.1. *Bayesian framework*

The Bayes' rule has frequently been employed to estimate the IIH map when the IIH is modeled by a distribution. Let $\beta$ be a random vector $(\beta_1, \dots, \beta_n)$ with probability density $p(\beta)$. To estimate $\beta$, one can maximize the conditional probability of $\beta$ given $\mathbf{y}$ (the log-transform of $\mathbf{x}$) as follows:

$$\widehat{\beta} = \max_{\beta} p(\beta \mid \mathbf{y}). \tag{4}$$

This is called the *maximum a posterior* (MAP) estimate and, by the Bayes rule, is equivalent to

$$\widehat{\beta} = \max_{\beta} p(\mathbf{y} \mid \beta) p(\beta). \tag{5}$$

Wells et al. [67] used the Gaussian distribution to model the entire log-transformed bias field and the observed intensity at voxel $i$:

$$p(\beta) = G_{\psi_\beta}(\beta), \tag{6}$$

$$p(y_i \mid \Gamma_i, \beta_i) = G_{\psi_{\Gamma_i}}(y_i - \mu(\Gamma_i) - \beta_i), \tag{7}$$

where $\Gamma_i$ is the tissue class at voxel $i$ with mean value $\mu(\Gamma_i)$, and

$$G_{\psi_{\mathbf{x}}}(\mathbf{x}) = (2\pi)^{-n/2} \left| \psi_{\mathbf{x}} \right|^{-1/2} \exp\left( -\frac{1}{2}\mathbf{x}^T \psi_{\mathbf{x}}^{-1} \mathbf{x} \right), \tag{8}$$

with $\psi_{\mathbf{x}}$ as the covariance matrix. By assuming the statistical independence of voxel intensities and from (7), one can derive

$$\begin{aligned} p(\mathbf{y} \mid \beta) &= \prod_i p(y_i \mid \beta_i) \\ &= \prod_i \sum_{\Gamma_i} p(y_i \mid \Gamma_i, \beta_i) p(\Gamma_i). \end{aligned} \tag{9}$$

When the image data is not polluted by IIH, the above method is simply the tissue classification using a mixture Gaussian model. Hence, this method essentially interleaves the IIH correction with a Gaussian classifier. Guillemaud and Brady [68] observed that the effect of IIH correction by Wells et al. algorithm is substantially affected by the Gaussian classifier. In real images, it is very possible for the histogram to deviate from the mixture Gaussian distribution. A modification was then proposed by introducing a tissue class $\Gamma_{\text{other}}$ with a non-Gaussian distribution

$$p(y_i \mid \beta_i) = \sum_{\Gamma_j} p(y_i \mid \Gamma_j) p(\Gamma_j) + \lambda p(\Gamma_{\text{other}}). \tag{10}$$

With this modification, the IIH is only estimated with respect to the Gaussian classes. Including the non-Gaussian component makes the Gaussian classifier less influenced by possible outliers arising in images.

### 4.2. Spatial modeling

In the method by Wells et al. the IIH correction has not explicitly considered the context of IIH map. Since the IIH field is slowly varying in the image domain, the values of the IIH map in neighboring pixels/voxels would be close. When this spatial relation is taken into account in the IIH modeling, one would likely arrive at a smoother approximation of the IIH map. A useful tool for spatial modeling is the Markov random field (MRF), which is first employed by Geman and Geman [72] for image segmentation. Held et al. [69] have employed the MRF to model IIH. According to the Hamersly-Clifford theorem [73], the prior probability $p(\mathbf{y})$ is given by the Gibbs distribution

$$p(\mathbf{y}) \propto \exp\{-U(\mathbf{y})\} \tag{11}$$

with the Gibbs energy

$$U(\mathbf{y}) = \alpha \sum_{\langle i,j\rangle} (y_i - y_j)^2 + \beta \sum_i y_i^2, \tag{12}$$

where $\langle i, j\rangle$ sums over every voxel $i$ and its neighbours $j$.

### 4.3. The N3 method

Different from most IIH correction method which involves a classification step, Sled et al. [74] proposed a nonparametric nonuniform intensity normalization (N3) method which searches for the IIH field to maximize the frequency content of the image intensity distribution. The method simplified the problem in log-domain as a deconvolution problem by realizing that if $v_1$ and $v_2$ are two independent random variables with distributions $V_1$ and $V_2$, respectively, then the distribution of their sum is the convolution of $V_1$ and $V_2$ [75]. To constrain the solution space, the IIH field is modeled as a Gaussian distribution with small variance. Code for this method is publicly available.[1]

### 5. OTHER APPROACHES

### 5.1. Comparison between local and global statistics

There are efforts [76, 77] to estimate the IIH by comparing a local statistic with the global one. The two statistics are assumed to characterize the same population. These methods essentially relate the IIH correction to tissue segmentation, and the implicit assumptions are (1) the constant intensity for a tissue and (2) that the intensity variation within a tissue is solely due to IIH. Not surprisingly, these methods are sensitive to the estimation of tissue statistics, which is nontrivial in practice.

When GM and WM are combined as one class and the local statistic is estimated from a sample in a local region as done in [76, 77], the method can be regarded as a generalized white matter method by Dawant et al. [48], where the reference tissue is the combination of GM and WM. Also, it can be taken as a lowpass filtering estimation. Although it is usually much easier to identify GM and WM together than to identify WM alone, a potential problem is that the local sample could fail to adequately characterize the feature of the combined tissue class even though there is no artifact like IIH.

A possible solution is to carry out more detail tissue classification in each local region. For example, the technique proposed in [78, 79][2] estimated the global tissue mean values by empirical thresholding, while the local statistics are derived through fitting the local histogram with a theoretical distribution. After obtaining estimations of local correction factors, a smooth function is fitted among these data and applied to the whole brain volume for IIH removal.

### 5.2. Image feature-based methods

An image feature-based technique was reported in [80], where the IIH correction was decomposed into row and column correction. The correction factor at a voxel is firstly related to first-order difference at other voxels in the same row/column, then combined with those calculated in the rows/columns from the initial to the current one. The rationale underlying the computation is obscure from the description. However, it is very similar to that in [81]. In the latter technique, a smooth "variation" image was firstly derived from normalized intensity gradient field, where pixels with low intensity or high gradient magnitude are excluded. Then numerical integration was applied to the "variation" (first-order derivative) image to obtain an image, which only contains small variations and was used to determine the IIH map.

Vovk et al. [82, 83] proposed to use the probability distribution of image features for IIH correction, where the image feature includes the intensity and the second spatial derivative of the image. Similar to the usual intensity histogram, the joint probability distribution also contains information for classifying tissues and such information was encoded by entropy. The correction factor was derived so that the entropy would decrease, similar to [57].

### 5.3. Estimate without explicit modeling

There are methods [84–88] which consider the IIH as model parameters formulated in a segmentation framework. Suppose the segmentation is to optimize a functional $\Phi(\mathbf{y}, \theta, \beta)$, where $\mathbf{y}$ denotes the observed data, $\beta$ the IIH term, and $\theta$

[1] http://www.bic.mni.mcgill.ca/software/N3

[2] Available with the BrainSuite package (http://neuroimage.usc.edu/).

other parameters. Then one way to estimate $\beta$ can be obtained by

$$\frac{\partial \Phi}{\partial \beta} = 0. \tag{13}$$

Rajapakse and Kruggel [84] used an MRF formulation, whereas Farag and his group exploited the fuzzy $c$-means clustering framework [85–88].

It is noted that in these methods no explicit assumption has been made on the IIH field, which can be advantageous over model-based methods, since the assumptions with a model could be violated in practice. On the other hand, the absence of constraints on the IIH solution could result in erroneous IIH maps that deviate far away from the truth. In addition, voxel-wise updating the IIH in an iterative process is time-consuming, hence techniques such as multigrid computing may help reduce the computation load.

### 5.4. *Registration against template*

Image registration has also been utilized to aid the IIH correction [89, 90]. In [90], the patient data was registered against a tissue reference template, which allows to estimate the IIH map by direct comparison between two images. Here human intervention was employed to ensure the correct correspondence between the distorted and the reference image.

### 5.5. *Shape recovery*

Lai and Fang [91] transformed the IIH correction into the problem of shape recovery with orientation constraint and solved the latter using regularization theory. The approach may result in solving a linear equation with a large matrix.

### 5.6. *Deformed thin plate model*

Bansal et al. [92] modelled the IIH field as a thin plate deforming elastically under a body force:

$$\mu \nabla^2 \beta + (\lambda + \mu)\nabla(\nabla \cdot \beta) + b(\beta) = 0, \tag{14}$$

where $\mu$ and $\lambda$ are the elasticity constants. The body force $b(\beta)$ is evaluated to minimize the entropy of the observed image.

### 6. DISCUSSION

### 6.1. *Integrated approaches*

As seen from the description above, many IIH correction methods relate the problem with image segmentation and solve these two problems alternatively through an iteration process. Evidently, accurate segmentation would significantly ease the burden of IIH correction. Conversely, if the IIH has been precisely removed, the segmentation accuracy will in general be improved. Thus, it is not surprising to see overwhelming techniques addressing these two problems within a common framework.

Actually, it has been a trend in computer vision to imitate the human intelligent system and solve the different problems simultaneously. A typical computer vision system may consist of several individual processes, which can be solved sequentially. However, the solution of one process could be beneficial to the solution of another one and the converse may also hold. As an example, image denoising and edge detection are two closely related problems. And it is common to require a denoising algorithm able to preserve image edge structures, and an edge detection method robust against noise. For the problem of IIH correction, beside the connection with image segmentation as mostly noted, there are efforts that relate the solution to image registration, because the image quality can impose an impact on the accuracy of image registration and conversely a good registration against a template could greatly help derive a high-quality image. In addition, there even exist attempts to address these three processes, IIH correction, segmentation, and registration together [93, 94].

Among some processes, their relationship may not be very intimate, but different implementation order could result in different performance. In [68], it was found that denoising after IIH correction is more preferable. Madabhushi and Udupa [95] investigated the interplay between IIH correction and intensity standardization, and concluded that the better sequence is IIH correction followed by intensity standardization.

### 6.2. *Validation and comparative study*

For end users, it is natural to ask questions such as how to assess the performance of an IIH correction method, which method should be recommended when a practical medical image processing system encounters the problem of IIH correction, or if there is a method which exclusively outperforms others. To answer these questions, we need to do extensive comparisons under a variety of data sets. However, this turns out to be a difficult task, because the true amount of IIH is unknown for real data. The lack of ground truth is a common problem in evaluating a computer vision algorithm. There are two possible ways to circumvent this difficulty. One is to approximate the golden standard by experts' estimation, which is often a tedious task. Alternatively, we can use synthetic data. In IIH correction, the simulated brain images [7, 96] from the Montreal Neurological Institute[3] have been widely employed for validation.

(1) *Criteria*

The criteria that have been frequently used are listed in the following.

Cr-I The variance of the fully or a partially segmented image, which is supposed to decrease after the IIH correction. When this criterion is used for comparing different methods, the result could be misleading

[3] http://www.bic.mni.mcgill.ca/brainweb because the variance is scale-variant. Usually, the mean-preserving condition is utilized to avoid this problem.

Cr-II The coefficient of variation, $cv$, of class $\Gamma_i$:

$$cv(\Gamma_i) = \frac{\sigma(\Gamma_i)}{\mu(\Gamma_i)}. \quad (15)$$

It can be shown that this quantity overcomes the limitation of the image variance. But the $cv$ alone only characterizes the within-class scatter and a criterion that also takes into account the between-class scatter is as follows.

Cr-III The coefficient of joint variations between two classes

$$cjv(\Gamma_1, \Gamma_2) = \frac{\sigma(\Gamma_1) + \sigma(\Gamma_2)}{|\mu(\Gamma_1) - \mu(\Gamma_2)|}. \quad (16)$$

Moreover, one can also use the relative change of $cjv$ as defined below:

$$\frac{cjv_a - cjv_b}{cjv_b} \times 100\%, \quad (17)$$

where the subscripts $a$ and $b$ denote after and before IIH correction.

Cr-IV Mean-square error, which directly measures the distance between the derived and the true IIH map.

Cr-V Segmentation accuracy, which indirectly reflects the effect of IIH correction. Care should be taken in interpreting the segmentation result since the latter could be complicated by other factors, like subject, scanner, noise, segmentation method, and so forth.

Cr-VI Stability, which means that an IIH correction algorithm is recursively applied to the corrected volume. For a good algorithm, the extracted IIH map is assumed to converge rapidly.

Cr-VII Computer requirement and CPU time.

From the list, one can observe that most criteria have their own limitations and some are applicable to simulated data only. However, simulated data might not adequately characterize real ones. For example, in [47], the proposed method was reported to be inferior to methods such as the N3 in terms of $cv$ or $cjv$ when tested on simulated data, but the order is reversed when tested against real volumes. Furthermore, the adaptivity of an algorithm is also important. For a method with good adaptivity, the approximated IIH map would approach a constant when the real IIH approaches zero.

(2) *Comparative study*

Compared to the numerous techniques for IIH correction, only a few studies have been carried out towards the comparative evaluation of existing algorithms. Sled et al. [97] have compared three IIH correction methods, the expectation maximization (EM) [67], the white matter (WM) [48], and the N3 method [74] using simulated T1, T2, and PD weighted data. It was shown that the WM method performs better than the other two methods for T1 weighted volumes, which might be due to the high contrast between the WM and other tissues in T1 weighted images. The EM method made excessively large corrections to voxels that fall outside the classifier's tissue model, as is consistent with that pointed out in [68]. Overall, the N3 method performs the most stable for all simulated images.

Velthuizen et al. [29] have evaluated four IIH correction methods (a phantom method [17], two lowpass filtering methods [36, 39], and a surface fitting method with reference points selected from white matter [48]) in brain tumor segmentation. The surface fitting method was found to be inferior to others, which could be due to the way the reference points were generated. As mentioned in Section 3.1, the latter is crucial to the performance of the surface fitting method. An automatic method to generate such reference points has been presented in [59]. Hou and Huang [98] have also developed a similar technique based on order statistics, which is very well comparable with the state-of-the-art IIH correction methods.

Although it turned out no improvement in tumor assessment after the IIH correction [29], it does not mean that IIH correction is not an obstacle to automatic medical image processing in general, since the tumor segmentation is characterized by the localization of the tumor region as well as the intensity contrast with surrounded tissue. Thus, the tumor segmentation could be less affected by the IIH artifact.

A more comprehensive study was presented in [66], where six algorithms, *n3* [74], *hum* [42], *eq* [43], *bfc* [78], *spm* (statistical parametric mapping)[4] [52], and *cma*[5] were compared against BrainWeb-simulated data as well as real volumes including repeated scans of the same subject, scans under different magnetic fields and different scanners. Three of the methods (*hum*, *eq*, and *cma*) are lowpass filtering based. The *spm* method is based on surface-fitting, and its parameters are estimated through integration with a tissue mixture model. It was found that the IIH maps obtained by filtering based methods can exhibit higher-frequency structures pertaining to brain anatomy. The *spm* method could be unstable when operating on relatively uniform image volumes and could lead to spurious solution for some volume. Overall, the *n3* and the *bfc* methods are superior to the other four methods. At lower bias levels, the estimated bias by *bfc* is more accurate than that by *n3*, and at higher bias levels, the order reverses. Nevertheless, none of the six methods performs ideally under all the circumstances investigated.

The problem of the *spm* might be similar to that of the EM method by Wells et al. [67]. Both methods utilized the mixture Gaussian classifier, which may be inadequate to model the image intensity distribution arising in practice. It should be pointed out that the *spm* method used in [66] is the SPM99 version, which has been updated to version SPM2 in 2003 with substantial improvement in theoretical modeling

[4] It is a part of the SPM99 software released by the Wellcome Department of Imaging Neuroscience (http://www.fil.ion.ucl.ac.uk/spm).

[5] Available in the Nautilos Library from the Center for Morphometric Analysis at the Massachusetts General Hospital.

or algorithmic design, and the latest version is SPM5. As to the three filtering-based methods, they lack a scheme to adapt the filtering strength to data quality, which may explain the inefficiency compared with the *n3* and the *bfc* methods. As mentioned in Section 2, filtering methods [45–47] with data adaptivity have been developed recently, which might outperform their conventional counterparts.

Although further comparative study using more extensive MR images is necessary, it might be inappropriate for end users to expect an algorithm superior to others and exclusively applicable. In general, each method has its underlying assumptions and limitations and the choice of which method to use is intimately interwined with the problem to solve, the source, and quality of the data. Many methods have attempted to correct the IIH artifact in brain MR images, some of which require the removal of the scalp/skull before the correction process, while others do not. Although sophisticated methods may be able to correct for IIH more accurately, one would also have to consider the expense of computer cost as well as the final segmentation error. Among the publicly available softwares, the N3 method has been widely used and its performance has been well demonstrated, while the BFC method can be advantageous when the image is also contaminated by severe noise [98].

### 7. CONCLUSION

This paper presented a summary of the recent progress on MR image IIH correction. The most popular models to describe the IIH field are the low frequency, the hypersurface, and the statistical model. Filtering methods are fast, easy to code and widely used. With optimization in scale space, the filtering method can also be adaptive to image data. Surface fitting and statistical methods are easy to integrate with other knowledge such as segmentation, registration, or some image feature, thus could in principle provide more reliable solution, which have been and will be the trend in the field. Some techniques based on other IIH correction principles were also reviewed in the paper. In future, it might be of interest to have more extensive investigations on evaluation of existing methods.

### REFERENCES

[1] G. H. Glover, C. E. Hayes, N. J. Pelc, et al., "Comparison of linear and circular polarization for magnetic resonance imaging," *Journal of Magnetic Resonance*, vol. 64, no. 2, pp. 255–270, 1985.

[2] I. Harvey, P. S. Tofts, J. K. Morris, D. A. G. Wicks, and M. A. Ron, "Sources of $T_1$ variance in normal human white matter," *Magnetic Resonance Imaging*, vol. 9, no. 1, pp. 53–59, 1991.

[3] A. Simmons, P. S. Tofts, G. J. Barker, and S. R. Arridge, "Sources of intensity nonuniformity in spin echo images at 1.5T," *Magnetic Resonance in Medicine*, vol. 32, no. 1, pp. 121–128, 1994.

[4] G. J. Barker, A. Simmons, S. R. Arridge, and P. S. Tofts, "A simple method for investigating the effects of non-uniformity of radiofrequency transmission and radiofrequency reception in MRI," *British Journal of Radiology*, vol. 71, no. 841, pp. 59–67, 1998.

[5] M. Alecci, C. M. Collins, M. B. Smith, and P. Jezzard, "Radio frequency magnetic field mapping of a 3 Tesla birdcage coil: experimental and theoretical dependence on sample properties," *Magnetic Resonance in Medicine*, vol. 46, no. 2, pp. 379–385, 2001.

[6] M. Styner, C. Brechbuhler, G. Szekely, and G. Gerig, "Parametric estimate of intensity inhomogeneities applied to MRI," *IEEE Transactions on Medical Imaging*, vol. 19, no. 3, pp. 153–165, 2000.

[7] D. L. Collins, A. P. Zijdenbos, J. G. Sled, N. J. Kabani, C. J. Holmes, and A. C. Evans, "Design and construction of a realistic digital brain phantom," *IEEE Transactions on Medical Imaging*, vol. 17, no. 3, pp. 463–468, 1998.

[8] P. A. Narayana, W. W. Brey, M. V. Kulkarni, and C. L. Sievenpiper, "Compensation for surface coil sensitivity variation in magnetic resonance imaging," *Magnetic Resonance Imaging*, vol. 6, no. 3, pp. 271–274, 1988.

[9] W. W. Brey and P. A. Narayana, "Correction for intensity falloff in surface coil magnetic resonance imaging," *Medical Physics*, vol. 15, no. 2, pp. 241–245, 1988.

[10] R. Stollberger and P. Wach, "Imaging of the active b1 field in vivo," *Magnetic Resonance in Medicine*, vol. 35, no. 2, pp. 246–251, 1996.

[11] P. J. Reber, E. C. Wong, R. B. Buxton, and L. R. Frank, "Correction of off resonance-related distribution in echo-planar imaging using EPI-based field maps," *Magnetic Resonance in Medicine*, vol. 39, pp. 328–330, 1998.

[12] K. R. Thulborn, F. E. Boada, G. X. Shen, J. D. Christensen, and T. G. Reese, "Correction of B1 inhomogeneities using echo-planar imaging of water," *Magnetic Resonance in Medicine*, vol. 39, no. 3, pp. 369–375, 1998.

[13] J. G. Sled and G. B. Pike, "Correction for $B_1$ and $B_0$ variation in quantitative $T_2$ measurements using MRI," *Magnetic Resonance in Medicine*, vol. 43, no. 4, pp. 589–593, 2000.

[14] A. Fan, W. W. Wells, J. W. Fisher, et al., "A unified variational approach to denoising and bias correction in MR," in *Proceedings of the International Conference on Information Processing in Medical Imaging (IPMI '03)*, C. J. Taylor and J. A. Noble, Eds., vol. 2732 of *Lecture Notes in Computer Science*, pp. 148–159, Ambleside, UK, July 2003.

[15] J. Wang, M. Qiu, Q. X. Yang, M. B. Smith, and R. T. Constable, "Measurement and correction of transmitter and receiver induced nonuniformities in vivo," *Magnetic Resonance in Medicine*, vol. 53, no. 2, pp. 408–417, 2005.

[16] B. R. Condon, J. Patterson, D. Wyper, A. Jenkins, and D. M. Hadley, "Image nonuniformity in magnetic resonance imaging: its magnitude and methods for its correction," *The British Journal of Radiology*, vol. 60, pp. 83–87, 1987.

[17] D. A. G. Wicks, G. J. Barker, and P. S. Tofts, "Correction of intensity nonuniformity in MR images of any orientation," *Magnetic Resonance Imaging*, vol. 11, no. 2, pp. 183–196, 1993.

[18] M. Ticher, C. R. Meyer, R. Gupta, and D. M. Williams, "Polynomial modeling and reduction of RF body coil spatial inhomogeneity in MRI," *IEEE Transactions on Medical Imaging*, vol. 12, no. 2, pp. 361–365, 1993.

[19] F. B. Mohamed, S. Vinitski, S. H. Faro, and C. F. Gonzalez, "Optimization of tissue segmentation of brain MR images based on multispectral 3d feature maps," *Magnetic Resonance Imaging*, vol. 17, no. 3, pp. 403–409, 1999.

[20] G. Collewet, A. Davenel, C. Toussaint, and S. Akoka, "Correction of intensity nonuniformity in spin-echo T1-weighted images," *Medical Resonance Imaging*, vol. 20, no. 4, pp. 365–373, 2002.

[21] E. R. McVeigh, M. J. Bronskill, and R. M. Henkelman, "Phase and sensitivity of receiver coils in magnetic resonance imaging," *Medical Physics*, vol. 13, no. 6, pp. 806–814, 1986.

[22] P. B. Roemer, W. A. Edelstein, C. E. Hayes, S. P. Souza, and O. M. Mueller, "The NMR phased array," *Magnetic Resonance in Medicine*, vol. 16, no. 2, pp. 192–225, 1990.

[23] D. C. Noll, C. H. Meyer, J. M. Pauly, D. G. Nishimura, and A. Macovski, "A homogeneity correction method for magnetic resonance imaging with time-varying gradients," *IEEE Transactions on Medical Imaging*, vol. 10, no. 4, pp. 629–637, 1991.

[24] C. E. Hayes, N. Hattes, and P. B. Roemer, "Volume imaging with MR phased arrays," *Magnetic Resonance in Medicine*, vol. 18, no. 2, pp. 309–319, 1991.

[25] S. E. Moyher, D. N. Vigneron, and S. J. Nelson, "Surface coil MR imaging of the human brain with an analytic reception profile correction," *Journal of Magnetic Resonance Imaging*, vol. 5, no. 2, pp. 139–144, 1995.

[26] C. M. Collins, Q. X. Yang, J. H. Wang, et al., "Different excitation and reception distributions with a single-loop transmit-receive surface coil near a head-sized spherical phantom at 300 MHz," *Magnetic Resonance in Medicine*, vol. 47, no. 5, pp. 1026–1028, 2002.

[27] B. P. Sutton, D. C. Noll, and J. A. Fessler, "Fast, iterative image reconstruction for MRI in the presence of field inhomogeneities," *IEEE Transactions on Medical Imaging*, vol. 22, no. 2, pp. 178–188, 2003.

[28] M. Markl, R. Bammer, M. T. Alley, et al., "Generalized reconstruction of phase contrast MRI: analysis and correction of the effect of gradient field distortions," *Magnetic Resonance in Medicine*, vol. 50, no. 4, pp. 791–801, 2003.

[29] R. P. Velthuizen, A. B. Cantor, H. Lin, L. M. Fletcher, and L. P. Clarke, "Review and evaluation of MRI nonuniformity corrections for brain tumor response measurements," *Medical Physics*, vol. 25, no. 9, pp. 1655–1666, 1998.

[30] R. C. Gonzalez and R. E. Woods, *Digital Image Processing*, Addison-Wesley, Singapore, 1993.

[31] D. Tomaževič, B. Likar, and F. Pernuš, "Comparative evaluation of retrospective shading correction methods," *Journal of Microscopy*, vol. 208, no. 3, pp. 212–223, 2002.

[32] J. Haselgrove and M. Prammer, "An algorithm for compensation of surface-coil images for sensitivity of the surface coil," *Magnetic Resonance Imaging*, vol. 4, no. 6, pp. 469–472, 1986.

[33] L. Axel, J. Constantini, and J. Listerud, "Intensity correction in surface coil MR imaging," *American Journal of Roentgenology*, vol. 148, no. 2, pp. 418–420, 1987.

[34] K. O. Lim and A. Pfefferbaum, "Segmentation of MR brain images into cerebrospinal fluid spaces, white and gray matter," *Journal of Computer Assisted Tomography*, vol. 13, no. 4, pp. 588–593, 1989.

[35] T. L. Jernigan, G. A. Press, and J. R. Hesselink, "Methods for measuring brain morphologic features on magnetic resonance images: validation and normal aging," *Archives of Neurology*, vol. 47, no. 1, pp. 27–32, 1990.

[36] P. A. Narayana and A. Borthakur, "Effect of radio frequency inhomogeneity correction on the reproducibility of intracranial volumes using MR image data," *Magnetic Resonance in Medicine*, vol. 33, no. 3, pp. 396–400, 1995.

[37] L. L. Wald, L. Carvajal, S. E. Moyher, et al., "Phased array detectors and an automated intensity-correction algorithm for high-resolution MR imaging of the human brain," *Magnetic Resonance in Medicine*, vol. 34, no. 3, pp. 433–439, 1995.

[38] J. W. Murakami, C. E. Hayes, and E. Weinberger, "Intensity correction of phased-array surface coil images," *Magnetic Resonance in Medicine*, vol. 35, no. 4, pp. 585–590, 1996.

[39] B. Johnston, M. S. Atkins, B. Mackiewich, and M. Anderson, "Segmentation of multiple sclerosis lesions in intensity corrected multispectral MRI," *IEEE Transactions on Medical Imaging*, vol. 15, no. 2, pp. 154–169, 1996.

[40] P. Irarrazabal, C. H. Meyer, D. G. Nishimura, and A. Macovski, "Inhomogeneity correction using an estimated linear field map," *Magnetic Resonance in Medicine*, vol. 35, no. 2, pp. 278–282, 1996.

[41] A. L. Reiss, M. T. Abrams, H. S. Singer, J. L. Ross, and M. B. Denckla, "Brain development, gender and IQ in children: a volumetric imaging study," *Brain*, vol. 119, no. 5, pp. 1763–1774, 1996.

[42] B. H. Brinkmann, A. Manduca, and R. A. Robb, "Optimized homomorphic unsharp masking for MR grayscale inhomogeneity correction," *IEEE Transactions on Medical Imaging*, vol. 17, no. 2, pp. 161–171, 1998.

[43] M. S. Cohen, R. M. Dubois, and M. M. Zeineh, "Rapid and effective correction of RF inhomogeneity for high field magnetic resonance imaging," *Human Brain Mapping*, vol. 10, no. 4, pp. 204–211, 2000.

[44] J. Luo, Y. Zhu, P. Clarysse, and I. Magnin, "Correction of bias field in MR images using singularity function analysis," *IEEE Transactions on Medical Imaging*, vol. 24, no. 8, pp. 1067–1085, 2005.

[45] C. Han, T. S. Hatsukami, and C. Yuan, "A multi-scale method for automatic correction of intensity non-uniformity in MR images," *Journal of Magnetic Resonance Imaging*, vol. 13, no. 3, pp. 428–436, 2001.

[46] F. Lin, Y. Chen, J. W. Belliveau, and L. L. Wald, "A wavelet-based approximation of surface coil sensitivity profiles for correction of image intensity inhomogeneity and parallel imaging reconstruction," *Human Brain Mapping*, vol. 19, no. 2, pp. 96–111, 2003.

[47] J. D. Gispert, S. Reig, J. Pascau, J. J. Vaquero, P. Garcia-Barreno, and M. Desco, "Method for bias field correction of brain T1-weighted magnetic resonance images minimizing segmentation error," *Human Brain Mapping*, vol. 22, no. 2, pp. 133–144, 2004.

[48] B. M. Dawant, A. P. Zijdenbos, and R. A. Margolin, "Correction of intensity variations in MR images for computer-aided tissue classification," *IEEE Transactions on Medical Imaging*, vol. 12, no. 4, pp. 770–781, 1993.

[49] C. R. Meyer, P. H. Bland, and J. Pipe, "Retrospective correction of intensity inhomogeneities in MRI," *IEEE Transactions on Medical Imaging*, vol. 14, no. 1, pp. 36–41, 1995.

[50] K. V. Leemput, F. Maes, D. Vandermeulen, and P. Suetens, "Automated model-based bias field correction of MR images of the brain," *IEEE Transactions on Medical Imaging*, vol. 18, no. 10, pp. 885–896, 1999.

[51] J. Ashburner and K. Friston, "Multimodal image coregistration and partitioning: a unified framework," *NeuroImage*, vol. 6, no. 3, pp. 209–217, 1997.

[52] J. Ashburner and K. Friston, "MRI sensitivity correction and tissue classification," *NeuroImage*, vol. 7, no. 4, part II, p. S706, 1998.

[53] J. Ashburner, *Computational neuroanatomy*, Ph.D. thesis, University College London, London, UK, 2000.

[54] J. Ashburner and K. J. Friston, "Voxel-based morphometry: the methods," *NeuroImage*, vol. 11, no. 6, part I, pp. 805–821, 2000.

[55] J. F. Mangin, "Entropy minimization for automatic correction of intensity nonuniformity," in *Proceedings of IEEE Workshop on Mathematical Methods in Biomedical Image Analysis*, pp. 162–169, Hilton Head Island, SC, USA, 2000.

[56] B. Likar, J. B. A. Maintz, M. A. Viergever, and F. Pernuš, "Retrospective shading correction based on entropy minimization," *Journal of Microscopy*, vol. 197, no. 3, pp. 285–295, 2000.

[57] B. Likar, M. A. Viergever, and F. Pernuš, "Retrospective correction of MR intensity inhomogeneity by information minimization," *IEEE Transactions on Medical Imaging*, vol. 20, no. 12, pp. 1398–1410, 2001.

[58] A. W.-C. Liew and H. Yan, "An adaptive spatial fuzzy clustering algorithm for 3-D MR image segmentation," *IEEE Transactions on Medical Imaging*, vol. 22, no. 9, pp. 1063–1075, 2003.

[59] D. Wang, S. E. Rose, J. B. Chalk, D. M. Doddrell, and J. Semple, "Improved version of white matter method for correction of non-uniform intensity in MR images: application to the quantification of rates of brain atrophy in Alzheimer's disease and normal aging," in *Proceedings of Medical Imaging 2000: Image Processing*, vol. 3979 of *Proceedings of SPIE*, pp. 760–771, San Diego, Calif, USA, February 2000.

[60] S. P. Liou, A. H. Chiu, and R. C. Jain, "A parallel technique for signal-level perceptual organization," *IEEE Transactions on Pattern Analysis and Machine Intelligence*, vol. 13, no. 4, pp. 317–325, 1991.

[61] P. K. Saha, J. K. Udupa, and D. Odhner, "Scale-based fuzzy connected image segmentation: theory, algorithms, and validation," *Computer Vision and Image Understanding*, vol. 77, no. 2, pp. 145–174, 2000.

[62] A. Madabhushi, J. K. Udupa, and A. Souza, "Generalized scale: theory, algorithms, and application to image inhomogeneity correction," in *Proceedings of Medical Imaging 2004: Image Processing*, vol. 5370 of *Proceedings of SPIE*, pp. 765–776, San Diego, Calif, USA, February 2004.

[63] S. Prima, N. Ayache, T. Barrick, and N. Roberts, "Maximum likelihood estimation of the bias field in MR brain images: investigating different modelings of the Imaging process," in *Proceedings of the 4th International Conference on Medical Image Computing and Computer-Assisted Intervention (MICCAI '01)*, vol. 2208 of *Lecture Notes in Computer Science*, pp. 811–819, Utrecht, The Netherlands, October 2001.

[64] A. Jannetta, J. C. Jackson, C. J. Kotre, I. P. Birch, K. J. Robson, and R. Padgett, "Mammographic image restoration using maximum entropy deconvolution," *Physics in Medicine and Biology*, vol. 49, no. 21, pp. 4997–5010, 2004.

[65] M. I. Reis and N. C. Roberty, "Maximum entropy algorithms for image reconstruction from projections," *Inverse Problems*, vol. 8, pp. 623–644, 1992.

[66] J. B. Arnold, J. Liow, K. A. Schaper, et al., "Qualitative and quantitative evaluation of six algorithms for correcting intensity nonuniformity effects," *NeuroImage*, vol. 13, no. 5, pp. 931–943, 2001.

[67] W. M. Wells III, W. E. L. Grimson, R. Kikinis, and F. A. Jolesz, "Adaptive segmentation of MRI data," *IEEE Transactions on Medical Imaging*, vol. 15, no. 4, pp. 429–442, 1996.

[68] R. Guillemaud and M. Brady, "Estimating the bias field of MR images," *IEEE Transactions on Medical Imaging*, vol. 16, no. 3, pp. 238–251, 1997.

[69] K. Held, E. R. Kops, B. J. Krause, W. M. Wells III, R. Kikinis, and H. M. Gartner, "Markov random field segmentation of brain MR images," *IEEE Transactions on Medical Imaging*, vol. 16, no. 6, pp. 878–886, 1997.

[70] Y. Zhang, M. Brady, and S. Smith, "Segmentation of brain MR images through a hidden Markov random field model and the expectation-maximization algorithm," *IEEE Transactions on Medical Imaging*, vol. 20, no. 1, pp. 45–57, 2001.

[71] J. L. Marroquin, B. C. Vemuri, S. Botello, F. Calderon, and A. Fernandez-Bouzas, "An accurate and efficient Bayesian method for automatic segmentation of brain MRI," *IEEE Transactions on Medical Imaging*, vol. 21, no. 8, pp. 934–945, 2002.

[72] S. Geman and D. Geman, "Stochastic relaxation, Gibbs distributions, and the Bayesian restoration of images," *IEEE Transactions on Pattern Analysis and Machine Intelligence*, vol. 6, no. 6, pp. 721–741, 1984.

[73] J. Besag, "Spatial interation and the statistical analysis of lattice systems," *Journal of the Royal Statistical Society B*, vol. 36, no. 2, pp. 192–225, 1974.

[74] J. G. Sled, A. P. Zijdenbos, and A. C. Evans, "Nonparametric method for automatic correction of intensity nonuniformity in MRI data," *IEEE Transactions on Medical Imaging*, vol. 17, no. 1, pp. 87–97, 1998.

[75] L. Devroye, *Non-Uniform Random Variate Generation*, Prentice-Hall, Englewood Cliffs, NJ, USA, 1986.

[76] S. K. Lee and M. W. Vannier, "Post-acquisition correction of MR inhomogeneities," *Magnetic Resonance in Medicine*, vol. 36, no. 2, pp. 275–286, 1996.

[77] C. DeCarli, D. G. M. Murphy, M. Tran, and D. Teichberg, "Local histogram correction of MRI spatially dependent image pixel intensity nonuniformity," *Journal of Magnetic Resonance Imaging*, vol. 6, no. 3, pp. 519–528, 1996.

[78] D. W. Shattuck, S. R. Sandor-Leahy, K. A. Schaper, D. A. Rottenberg, and R. M. Leahy, "Magnetic resonance image tissue classification using a partial volume model," *NeuroImage*, vol. 13, no. 5, pp. 856–876, 2001.

[79] D. W. Shattuck and R. M. Leahy, "BrainSuite: an automated cortical surface identification tool," *Medical Image Analysis*, vol. 6, no. 2, pp. 129–142, 2002.

[80] A. Koivula, J. Alakuijala, and O. Tervonen, "Image feature based automatic correction of low-frequency spatial intensity variations in MR images," *Magnetic Resonance Imaging*, vol. 15, no. 10, pp. 1167–1175, 1997.

[81] E. A. Vokurka, N. A. Thacker, and A. Jackson, "A fast model independent method for automatic correction of intensity nonuniformity in MRI data," *Journal of Magnetic Resonance Imaging*, vol. 10, no. 4, pp. 550–562, 1999.

[82] U. Vovk, F. Pernuš, and B. Likar, "Multi-feature intensity inhomogeneity correction in MR images," in *Proceedings of the 7th International Conference on Medical Image Computing and Computer-Assisted Intervention (MICCAI '04)*, pp. 283–290, Saint-Malo, France, September 2004.

[83] U. Vovk, F. Pernuš, and B. Likar, "MRI intensity inhomogeneity correction by combining intensity and spatial information," *Physics in Medicine and Biology*, vol. 49, no. 17, pp. 4119–4133, 2004.

[84] J. C. Rajapakse and F. Kruggel, "Segmentation of MR images with intensity inhomogeneities," *Image and Vision Computing*, vol. 16, no. 3, pp. 165–180, 1998.

[85] D. L. Pham and J. L. Prince, "Adaptive fuzzy segmentation of magnetic resonance images," *IEEE Transactions on Medical Imaging*, vol. 18, no. 9, pp. 737–752, 1999.

[86] D. L. Pham and J. L. Prince, "An adaptive fuzzy c-means algorithm for image segmentation in the presence of intensity inhomogeneities," *Pattern Recognition Letters*, vol. 20, no. 1, pp. 57–68, 1999.

[87] M. N. Ahmed, S. M. Yamany, N. A. Mohamed, and A. A. Farag, "A modified fuzzy c-means algorithm for MRI bias-field estimation and adaptive segmentation," in *Proceedings of the International Conference on Medical Image Computing and Computer-Assisted Intervention (MICCAI '99)*, pp. 72–81, Cambridge, UK, September 1999.

[88] M. N. Ahmed, S. M. Yamany, N. Mohamed, A. A. , Farag, and T. Moriarty, "A modified fuzzy c-means algorithm for bias field estimation and segmentation of MRI data," *IEEE Transactions on Medical Imaging*, vol. 21, no. 3, pp. 193–199, 2002.

[89] K. J. Friston, J. Ashburner, C. D. Frith, J. D. Heather, J. B. Poline, and R. S. J. Frackowiak, "Spatial registration and normalization of images," *Human Brain Mapping*, vol. 3, no. 3, pp. 165–189, 1995.

[90] C. Studholme, V. Cardenas, E. Song, F. Ezekiel, A. Maudsley, and M. Weiner, "Accurate template-based correction of brain MRI intensity distortion with application to dementia and aging," *IEEE Transactions on Medical Imaging*, vol. 23, no. 1, pp. 99–110, 2004.

[91] S. H. Lai and M. Fang, "A new variational shape-from-orientation approach to correcting intensity inhomogeneities in magnetic resonance image," *Medical Image Analysis*, vol. 3, no. 4, pp. 409–424, 1999.

[92] R. Bansal, L. H. Staib, and B. S. Peterson, "Correcting nonuniformities in MRI intensities using entropy minimization based on an elastic model," in *Proceedings of the 7th International Conference on Medical Image Computing and Computer-Assisted Intervention (MICCAI '04)*, pp. 78–86, Saint-Malo, France, September 2004.

[93] B. Fischl, D. H. Salat, E. Busa, et al., "Whole brain segmentation: automated labeling of neuroanatomical structures in the human brain," *Neuron*, vol. 33, no. 3, pp. 341–355, 2002.

[94] J. Ashburner and K. J. Friston, "Unified segmentation," *NeuroImage*, vol. 26, no. 3, pp. 839–851, 2005.

[95] A. Madabhushi and J. K. Udupa, "Interplay between intensity standardization and inhomogeneity correction in MR image processing," *IEEE Transactions on Medical Imaging*, vol. 24, no. 5, pp. 561–576, 2005.

[96] C. Cocosco, V. Kollokian, R. S. Kwan, and A. Evans, "Brain web: online interface to a 3D MRI simulated brain database," *NeuroImage*, vol. 5, no. 4, part II, p. S425, 1997.

[97] J. G. Sled, A. P. Zijdenbos, and A. C. Evans, "A comparison of retrospective intensity non-uniformity correction methods for MRI," in *Proceedings of the15th International Conference in Information Processing in Medical Imaging*, vol. 1230 of *Lecture Notes in Computer Science*, pp. 459–464, Poultney, Vt, USA, June 1997.

[98] Z. Hou and S. Huang, "Preliminary segmentation based on order statistics for intensity inhomogeneity correction in brain MR images," Technique Report, Singapore Bioimaging Consortium, Singapore, 2002, available upon request.

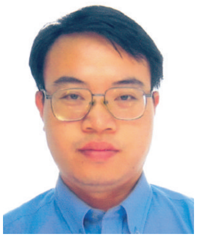

**Zujun Hou** is with Singapore Bioimaging Consortium as a Research Scientist. His research interest has evolved from ecology and bioremediation, chaotic system dynamics, to image processing and pattern recognition, with current focus on medical image processing and biometrics. He received the B.S. degree from the Department of Physics in Beijing Normal University, China, in 1991, the M.S. and the Ph.D. degrees from the Department of Physics and the Department of Computational Science in National University of Singapore, in 1999 and 2003, respectively.

